# Adding a quadrivalent human papillomavirus vaccine to the UK cervical cancer screening programme: A cost-effectiveness analysis

**DOI:** 10.1186/1478-7547-6-4

**Published:** 2008-02-15

**Authors:** Shalini L Kulasingam, Steve Benard, Ruanne V Barnabas, Nathalie Largeron, Evan R Myers

**Affiliations:** 1 Dept. of Obstetrics and Gynecology, Duke University, Durham, NC 27705, USA; 2 sanofi pasteur MSD, Lyon, 69007, France; 3 Cancer Epidemiology Unit, University of Oxford, Oxford, OX3 7LF, UK; 4 HIV Vaccines Trials Network, Fred Hutchinson Research Center, Seattle, WA, USA

## Abstract

**Background:**

We assessed the cost-effectiveness of adding a quadrivalent (6/11/16/18) human papillomavirus (HPV) vaccine to the current screening programme in the UK compared to screening alone.

**Methods:**

A Markov model of the natural history of HPV infection incorporating screening and vaccination was developed. A vaccine that prevents 98% of HPV 6, 11, 16 and 18-associated disease, with a lifetime duration and 85% coverage, in conjunction with current screening was considered.

**Results:**

Vaccination with screening, compared to screening alone, was associated with an incremental cost-effectiveness ratio of £21,059 per quality adjusted life year (QALY) and £34,687 per life year saved (LYS). More than 400 cases of cervical cancer, 6700 cases of cervical intraepithelial neoplasia and 4750 cases of genital warts could be avoided per 100,000 vaccinated girls. Results were sensitive to assumptions about the need for a booster, the duration of vaccine efficacy and discount rate.

**Conclusion:**

These analyses suggest that adding a quadrivalent HPV vaccine to current screening in the UK could be a cost-effective method for further reducing the burden of cervical cancer.

## Background

Despite a well-organised screening programme in the UK, and a marked decrease in cervical cancer incidence since 1988, there were 3,181 new cervical cancer cases and 1,529 deaths reported in 2002. In 2003, the National Health Service Cervical Screening Programme modified its recommendations by increasing the age to begin screening from 20 years to 25 years combined with a more frequent screening interval (every 3 years in women aged 25 to 49 years and 5 years for women between 50 and 64).

Invasive carcinoma of the cervix is preceded by premalignant lesions. These precancerous lesions are defined as cervical intraepithelial neoplasia (CIN), and classified as low grade (CIN 1) or high grade (CIN 2 or CIN 3) according to severity. Prevention of cervical cancer has been based on early detection of these precancerous lesions using conventional Pap smear tests or, more recently, liquid-based cytology (LBC) tests. However, with the knowledge that infection with oncogenic human papillomavirus (HPV) is necessary for the development of cervical cancer [1], alternative methods, beside the Pap smear are being researched to improve cervical cancer prevention. In 2006, the first prophylactic quadrivalent HPV recombinant vaccine (HPV types 6,11,16,18) (Gardasil^®^, Merck, Sharpe and Dohme (MSD), Whitehouse Station, New Jersey, USA) has been granted a marketing authorisation in the European Union [2]. This vaccine is indicated for the prevention of high grade cervical dysplasia (CIN 2/3), cervical carcinoma, high grade vulvar dysplastic lesions (VIN 2/3) and external genital warts causally related to HPV types 6, 11, 16 and 18. More recently, the European Commission has granted a marketing authorisation for a second cervical cancer vaccine (Cervarix, GlaxoSmithKline Biologicals s.a., Rixensart, Belgium) that is indicated for the prevention of precancerous cervical lesions (high-grade cervical intraepithelial neoplasia [CIN] grades 2 and 3) and cervical cancer causally related to human papillomavirus (HPV) types 16 and 18 [3]. Although the UK Health Minister has recommended the use of the HPV vaccine for girls aged 12–13 and catch-up for girls aged up to 19 years, a decision has not yet been made regarding which vaccine to use in the National Immunization program [4].

The quadrivalent vaccine showed >90% efficacy in preventing pre-cancerous high grade lesions due to these two HPV types [5,6]. This vaccine presents an opportunity to further reduce cancer incidence and mortality.

Genital warts are the most common sexually transmitted infection in the UK. In 2004, 79,678 first attack cases of genital warts were reported in Genitourinary Medicine (GUM) clinics [7]. Of these, 47% were diagnosed in women and 53% in men. Current methods for treating warts include therapies such as cryotherapy, electrocautery, podophyllotoxin and imiquimod. However, up to 40% of patients experience a recurrence of genital warts post-treatment [8]. The psychological impact of warts can be high; both men and women report feelings of embarrassment and depression [9]. Over 90% of genital warts are attributable to infection with HPV types 6 and 11 [10]. Results from a Phase III trial of a quadrivalent vaccine that includes HPV types 6 and 11, in addition to the oncogenic HPV types 16 and 18, showed that vaccination prevented >90% of warts [11].

We examined the potential effectiveness and cost-effectiveness of a quadrivalent vaccine targeted at HPV types 6, 11, 16 and 18, administered to a cohort of girls aged 12 through a school-based vaccination programme in conjunction with the current screening programme in the UK over a lifetime period.

## Methods

We adapted a previously published and validated state-transition Markov model of HPV infection and cervical cancer [12,13] to estimate total lifetime costs, life expectancy and incremental cost-effectiveness ratios (ICERs) associated with different screening strategies either alone or in combination with vaccination to prevent HPV types 6, 11, 16 and 18 in the UK. Estimates and ranges used in the model for the natural history are presented in Table 1. Briefly, the model simulates a cohort of women at age 12 and follows them until age 85 years. Movement through the health states of the model (i.e. HPV infection, CIN 1, CIN 2, CIN 3, Cancer [Stages I–IV]) over time is based on yearly transition probabilities derived from the literature. Women who are infected with HPV can have their infection clear, progress, or persist. For those women whose infections persist, the majority is assumed to develop CIN 1 but a minority is assumed to develop CIN 2 directly; these rates are age-dependant. Women who develop CIN 1, CIN 2, or CIN 3 can have their disease progress, regress, or persist. Women with cancer (stages I, II, III, IV) can have their cancer detected during screening or if they present to a health care provider based on symptoms. Women who do not have their disease detected can progress to the next stage, remain in the same stage, or die of cervical cancer. Each year, women also face an age-specific risk of dying from other causes.

**Table 1:** Annual transition probabilities for the natural history model

**Parameters**	**Age**	**Transition probability**	**Time period**	**References**
***Normal***				
Uninfected to Cervical HPV infection (HPV incidence)	10–12	0.0000	12 months	Calibrated from Canfell et al^17^
	13	0.0100	12 months	
	14	0.0300	12 months	
	15	0.0400	12 months	
	16	0.0460	12 months	
	17	0.0700	12 months	
	18	0.0700	12 months	
	19	0.1700	12 months	
	20–21	0.2000	12 months	
	22	0.1200	12 months	
	23	0.1100	12 months	
	24–29	0.0850	12 months	
	30–33	0.0320	12 months	
	34–49	0.0170	12 months	
	50+	0.0095	12 months	
				
***HPV infected state***				
Progression from HPV infection to SIL – all risk HPV		0.0959	12 months	Canfell et al^17^
Percentage CIN 2 among SIL		0.1350	12 months	Calibrated based on Myers et al^12 ^and Canfell et al^17^
Regression of CIN 1 to normal from HPV infection	12–24	0.7000	18 months	Calibrated based on Myers et al^12 ^and Canfell et al^17^
	25–29	0.5000	18 months	
	30–39	0.4000	18 months	
	40–49	0.2700	18 months	
	50+	0.1000	18 months	
				
***CIN 1***				Canfell et al^17^
Progression from CIN 1 to CIN 2 – all risk HPV	16–34	0.0297	12 months	
	35+	0.1485	12 months	
Progression from CIN 1 to CIN 3 – all risk HPV		0.0301	12 months	
Regression to HPV infected state – all risk HPV	16–34	0.2248	12 months	
	35+	0.1124	12 months	
Proportion regressing to normal		0.9000	12 months	
				
**CIN 2**				Canfell et al^17^
Progression from CIN 2 to CIN 3	16–34	0.0389	12 months	
	35–44	0.0797	12 months	
	45+	0.1062	12 months	
Regression from CIN 2 to CIN 1		0.2430	12 months	
Regression from CIN 2 to uninfected or HPV infections		0.1901	12 months	
Proportion regressing directly to normal		0.9000	12 months	
				
***CIN 3***				Canfell et al^17^
Regression CIN 3 to CIN 1 – all risk HPV		0.0000	12 months	
Regression from CIN 3 to CIN 2 – all risk HPV		0.0135	12 months	
CIN 3 to uninfected or HPV infection	16–44	0.0135	12 months	
	45+	0.0100	12 months	
Proportion CIN 3 regressing directly to uninfected		0.5000	12 months	
Proportion CIN 3 progressing to FIGO I cancer		0.0127	12 months	
				
**Cervical cancer**				Myers et al^12^
FIGO 1				
Progression rates		0.9000	48 months	
Probability of symptoms		0.1850	12 months	
FIGO 2				
Progression rates		0.9000	36 months	
Probability of symptoms		0.3000	12 months	
FIGO 3				
Progression rates		0.9000	15 months	
Probability of symptoms		0.7500	12 months	
FIGO 4				
Probability of symptoms		0.8000	12 months	
				
Annual probability of survival after diagnosis, FIGO 1				Cancer Research UK ^21^
1 Year survival		0.977	12 months	
2 Year survival		0.978	12 months	
3 Year survival		0.963	12 months	
4 Year survival		0.988	12 months	
5 Year survival		0.988	12 months	
				
Annual probability of survival after diagnosis, FIGO 2				
1 Year survival		0.830	12 months	
2 Year survival		0.835	12 months	
3 Year survival		0.755	12 months	
4 Year survival		0.870	12 months	
5 Year survival		0.899	12 months	
				
Annual probability of survival after diagnosis, FIGO 3				
1 Year survival		0.590	12 months	
2 Year survival		0.693	12 months	
3 Year survival		0.778	12 months	
4 Year survival		0.928	12 months	
5 Year survival		0.963	12 months	
				
Annual probability of survival after diagnosis, FIGO 4				
1 Year survival		0.523	12 months	
2 Year survival		0.782	12 months	
3 Year survival		0.721	12 months	
4 Year survival		0.925	12 months	
5 Year survival		0.956	12 months	

The model was calibrated to produce prevalence curves for HPV infection [14,15], cervical cancer lifetime risks and cervical cancer incidence in the UK [16]. The model was revised to separate high-grade CIN into CIN 2 and CIN 3 using data from Canfell et al. [17]. Non-cervical cancer deaths were estimated using data from UK statistics [18]. Benign hysterectomy rates were estimated using age-specific estimates from Redburn et al. [19]. Cancer progression rates between FIGO (International Federation of Gynecology and Obstetrics) stages (FIGO I through IV) were based on the original model [12]. Cancer stage-specific symptoms were based on calibrating the model to produce a stage-specific distribution of cancer, in the absence of screening consistent with Bjorge et al. [20]. Five-year stage-specific survival was based on data from the West Midlands [21]. Finally, we assumed that only women who were normal (i.e. did not have CIN or cervical cancer) were at risk for developing warts due to a lack of published data on women who have CIN or cancer and warts. We used data from the Health Protection Agency [7] to determine the "incidence" of symptomatic warts, since these data are based on women presenting to clinics with symptoms. We conservatively assumed that all women with symptomatic warts would receive treatment and that treatment was 100% effective.

For the base case, women aged 25 to 49 years were assumed to be screened every 3 years; women aged 50 to 64 years were screened every 5 years consistent with current National Guidelines [22]. Differences in screening coverage by age were modelled using estimates from the Government Statistical Service (2003). Estimates for the sensitivity and specificity of conventional cytology and liquid cytology tests were based on published data [23,24] and UK specific data [25], with separate estimates of sensitivity used for CIN 1/CIN 2 and CIN 3. Fifty percent of women were assumed to be screened with LBC and the rest were assumed to be screened with conventional Pap smears for the base case. Ten percent of women were estimated to have inadequate Pap smear screening results and were assumed to undergo repeat screening [26]. Women with normal Pap smear results were assumed to return to regular screening. Women with Atypical Squamous Cells with Unknown Significance (ASCUS) or Low grade Squamous Intraepithelial Lesion (LSIL) Pap smear results were assumed to undergo repeat screening, with women referred to colposcopy based on two repeat borderline results. Women with ≥ High grade Squamous Intraepithelial Lesion (HSIL) were assumed to be referred directly to colposcopy. Colposcopy and biopsy were assumed to have 90% sensitivity for detection of CIN [27]. Treatment of CIN was assumed to be 100% effective. Twenty percent of women with CIN 1 were assumed to be treated: this proportion is consistent with the recommendation that confirmed CIN 1 lesions are monitored via colposcopy rather than treated [17]. The proportion of women treated for CIN 2 and 3 was assumed to be 90% [17]. Screening and treatment parameters are presented in Table 2.

**Table 2:** Screening, vaccine and cost parameters

**Parameters**	**Base case**	**Ranges**	**References**
**Screening characteristics**			
Screening interval	3 years in ages 25–49 years and 5 years in ages 50–64 years		NHS cervical screening programme ^22^
Coverage rates of target groups by age (2003)			
25–29	74.0%		NHS cervical screening programme ^22^
30–34	81.0%		
35–39	83.7%		
40–44	84.0%		
45–49	83.8%		
50–54	83.2%		
55–59	81.4%		
60–64	77.3%		
Inadequate pap smear results	10%	5% – 20%	
Pap Sensitivity for CIN 1/2 Pap sensitivity for CIN 1/2 (LBC)	61%	51% – 80%	Nanda et al^23^and Karnon et al^25^
Pap Sensitivity for CIN 3 Pap Sensitivity for CIN 3 (LBC)	65%	65% – 90%	Nanda et al^23 ^and Karnon et al^25^
Pap Specificity for no CIN Pap Specificity for no CIN (LBC)	95.7%	90% – 99%	Nanda et al^25 ^and Kulasingam et al^24^
Colposcopy/Biopsy Sensitivity	90%	88% – 100%	Mitchell et al^27^
Colposcopy/Biopsy Specificity	100%	65% – 100%	Kulasingam et al^24 ^and Karnon et al^25^
***Vaccine characteristics***			
Vaccine efficacy for all 6, 11, 16, 18 HPV types	98%	85% – 100%	Villa et al^5 ^and Future II ^6^
Duration of efficacy	Lifetime	From 10 years to lifetime	Olsson et al ^28 ^and Villa et al^42^
Vaccine coverage	85%	50%–90%	Trotter et al^31 ^and Bramley et al^32^
Booster coverage		50%	Trotter et al^31^
**Costs**			
Pap smear	£23.7	£18 – £30	Brown et al ^26^ Curtis et al^38^
Colposcopy (with or without biopsy)	£141.69	£113 – £170	
Knife cone biopsy of cervix uteri	£290.64	£232 – £349	
CIN 1, CIN 2, CIN 3	£313.14	£250 – £376	
FIGO I	£12,142	£9,714 – £14,570	Curtis et al^38 ^and Wolstenholme et al^37^
FIGO II	£22,061	£17,649 – £26,473	
FIGO III	£21,785	£17,428 – £26,142	
FIGO IV	£23,402	£18,722 – £28,082	
Genital warts	£215.73	£172 – £259	Brown et al^26 ^and Curtis et al^38^
Vaccine cost/dose	£75	£70 – £80	
Administration cost/dose	£ 3.4	£0 – £12	Trotter et al^31^
Booster cost/dose		£75	
Administration cost for booster		£10	Curtis et al^38^
**Discount rates**			
Costs	3.5%	0 – 5%	
Benefits	3.5%	0 – 5%	

Vaccination to prevent infection with HPV types 6, 11, 16 and 18 was assumed to be 98% effective, using the recent results from the FUTURE II trial to determine efficacy of the vaccine in preventing CIN 2–3 [6]. We conservatively assumed the same efficacy for genital warts [5], but varied this assumption widely in sensitivity analyses. The vaccine was assumed to be administered to girls aged 12 years through a school-based programme. To date, there is evidence of a 5-year duration of vaccine efficacy [28]. We assumed a lifetime duration of efficacy for the base case consistent with a recently published analysis of the impact of an HPV 16–18 vaccine on cervical cancer in the UK [29] as well as other analyses [30] but varied this assumption widely in sensitivity analyses. Use of a booster, assumed to be administered 10 years after the initial vaccine (i.e., at age 22), to achieve a lifetime duration of efficacy was examined in a sensitivity analysis [31]. Vaccine coverage was 85% for the base case based on coverage rates reported for the hepatitis B vaccine in the UK through a school programme [32]. Since women can be infected with multiple HPV types, and these other types can potentially cause replacement CIN and cancer, we examined this possibility in sensitivity analyses, assuming that 10 percent of women were coinfected with other high-risk HPV types [33].

We modelled a reduction of approximately 35% for CIN 1, 55% for CIN 2 and 3 and 70% for cervical cancer (all stages). This reflects the percentage of cervical cancer and CIN 1–3 attributable to HPV types 6, 11, 16 and 18 [34,35]. Moreover, we assumed that 90% of warts were attributable to infection with HPV types 6 and 11 [10]. We modelled the impact of the vaccine as a direct reduction in CIN rather than developing a type-specific model to account for reductions in HPV type-specific infection, taking into account type-specific progression and regression through the different CIN and cancer states similar to Goldie et al. [36].

Costs for screening, diagnosis and treatment for cervical cancer as well as for diagnosing and treating warts were obtained from previously published studies [37,38] and are presented in Table 2. The costs were inflated to 2005 £ using the Hospital and Community Services pay and prices index [38]. While the NHS price of the vaccine is £80.50, a volume-based discount will be applied for any vaccination programme. For the purposes of this analysis, we have assumed a cost per dose for the vaccine of £75 was used but varied from £70 to £80 in sensitivity analyses. The cost for administration was assumed to be £3.40 per dose for the base case, but varied up to £12 in sensitivity analyses. Only direct costs were included in the analyses, assuming a National Health System (NHS) perspective.

Utilities for calculating quality-adjusted life expectancy were based on ongoing studies and are summarized in Table 3[39,40]. Time with disease was based on expert opinion (Dr Barnabas, personal communication, 2005). The utility value for those surviving cervical cancer was assumed to be 1.0

**Table 3:** Utility scores

**Parameters**	**Utility**	**Time with Disease**	**Ranges**	**References**
Screening Pap	0.98	1 months	2 weeks – 2 months	Myers et al^39 ^and Insinga et al^40^
ASCUS pap	0.94	1 month	2 weeks-2 months	
>= LSIL pap	0.91	2 months	1–4 months	
Warts	0.91	2 months	1–4 months	
CIN 1	0.91 0.96	2 months 10 months	2–4 months 0–10 months	
CIN 2–3	0.87	2 months	1–4 months	
FIGO I	0.76	5 years	1–5 years	
FIGO II	0.67	5 years	1–5 years	
FIGO III	0.67	5 years	1–5 years	
FIGO IV	0.67	5 years	1–5 years	

Health outcomes and costs are discounted at 3.5% annually for the base case. Results are presented as average lifetime costs, average life-expectancy, life year saved (LYS), Quality adjusted life year (QALY) and incremental cost-effectiveness ratios (ICERs). Strategies that were more costly and less effective, or less cost-effective than adjacent strategies were considered "dominated."

## Results

### Validation of the model

**Figure 1:**
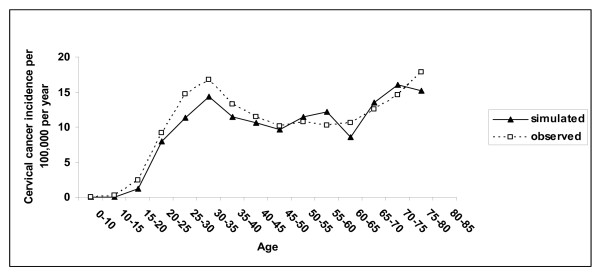
**Observed and predicted incidence of invasive cervical cancer in the UK.** UK statistics. Cancer registration in England, 2002.

The predicted age-specific annual incidence of invasive cervical cancer in the UK population is similar to the observed data in the UK (Figure 1). The model predicts a lifetime risk of cancer in the absence of screening, for women aged 20 to 79 years, of 2.0% and 0.71% with screening, which are similar to estimates from a previously published modelling study that examined the impact of changes in recommendations to the UK screening programme [17]. The distribution for FIGO stages predicted by the model is similar to data reported by Bjorge et al. [20] (Stage I: 56%, Stage II: 29%, Stage III: 12%, Stage IV: 3%).

### Clinical outcomes

When vaccination is added to screening, under base case assumptions, the lifetime risk of cancer is reduced from 0.71 to 0.29%. Considering a cohort of 100,000 women in the UK, the model estimates that around 418 cervical cancers, 127 deaths, 2,554 CIN 1, 1,683 CIN 2, 2,479 CIN 3 and 4,798 genital warts could be avoided (Table 4).

**Table 4:** Estimated cases of cervical cancer, cervical cancer deaths, cervical intraepithelial neoplasia grade 1 (CIN 1), grade 2 (CIN 2), grade 3 (CIN 3) and genital warts cases per 100 000 women who are screened, or vaccinated and screened over a lifetime

	**Cervical cancer cases**	**Deaths from cervical cancer**	**CIN 3 cases detected**	**CIN 2 cases detected**	**CIN 1 cases detected**	**Genital warts cases**
**Screening only**	715	218	5325	3906	12453	7147
**Screening and vaccination**	297	91	2846	2223	9899	2349
**Case avoided**	**418**	**127**	**2479**	**1683**	**2554**	**4798**

* 85% vaccine coverage rate and lifetime duration of vaccine efficacy

### Economic outcomes

**Figure 2:**
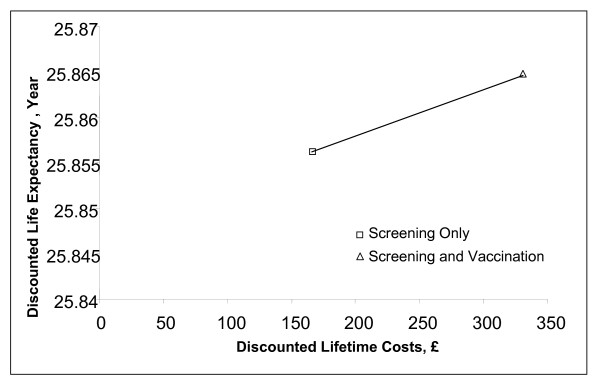
Efficiency curve comparing a strategy of screening only to a strategy of vaccination plus screening.

Compared to no screening or vaccination (natural history), screening only is associated with an ICER of £11,156 per QALY. Compared to screening, vaccination combined with screening had an incremental cost-effectiveness ratio (ICER) of £21,059 per QALY and £34,687 per LYS (Figure 2 and Table 5).

**Table 5:** One-way sensitivity analyses comparing cervical cancer screening only and cervical cancer screening associated with a quadrivalent HPV vaccination programme

**Parameters**	**ICER (£/QALYs)**	**ICER (£/LYs)**
**Base case**	21,059	34,687
**Vaccine duration**		
10 years	68,417	116,743
10 years + booster to achieve lifetime protection	26,782	44,114
20 years	30,777	52,578
**Multiple infections**		
15%	24,085	39,842
**Vaccine efficacy**		
85%	25,081	40,831
**Vaccine coverage**		
**50%**	**21,581**	**34,426**
**Screening coverage rate**		
-50%	16,266.	35,476
-10%	19,926	34,681
**Screening, diagnosis and treatment costs**		
-20%	21,717	35,771
+20%	20,401	33,602
**Vaccine costs**		
70 £	19,450	32,036
80 £	22,668	37,337
**Utilities**		
25% decrease for screening utilities; 1-year duration for time with cancer	25,600	
25% increase in time with disease; 5-year duration for time with cancer	19,840	
Cancer utilities only (5 year duration)	27,954	
**Discount rates**		
0% costs; 0% medical benefit	3,123	4,122
3% costs; 3% medical benefit	17,089	27,066
3,5% costs; 1,5% medical benefit	9,653	13,797
5% costs; 5% medical benefit	36,618	68,760
**Multivariate Sensitivity Analyses**		
10 years duration, 50% coverage, 85% efficacy	84,925	140,705
Lifetime duration, 90% coverage, 100% efficacy	20,316	33,752
**Changes to Screening (assuming base case assumptions for the vaccine)**		
Screening every 5 years starting at age 25	13,449	36,712
Screening starting at age 26	20,724	34,441
Screening starting at age 28	16,527	34,153
Screening starting at age 30	13,680	34,989

Base case discount rate: 3.5% for costs and medical benefits

As shown in Table 5, results were sensitive to the assumption used for the duration of efficacy. If a 10 year duration of vaccine efficacy is assumed, the ICER for screening and vaccination compared to screening only would be £68,417 per QALY (£116,743 per LY). If a booster was needed to achieve lifetime protection, the ICER was £26,782 per QALY (£44,114 per LY) under the assumption that the booster was given at age 22 and coverage at that age was 50%. Results were moderately sensitive to time with an abnormality with ICERs for screening and vaccination compared to screening only ranging from £19,840 (for a 25% increase in the length of time with abnormality) to £25,699 (for a 25% decrease in the length of time) when screening, diagnosis and cancer were varied. Results were very sensitive to the discount rate considered for benefits: varying discount rates from 3,5% for medical benefits (base case) to 1,5% would decrease the ICER to £9,653 per QALY.

Varying the costs for screening, diagnosis and treatment (Table 2) over a wide range, as well as varying the cost of the vaccine between £70 and £80 had a moderate impact on the cost-effectiveness of screening and vaccination compared to screening only.

In multi-variable sensitivity analyses, we used a best and worst case scenario to determine the possible bounds for key aspects of the vaccine (duration, coverage and efficacy). As shown, a combination of lower coverage, lower efficacy and short duration has an important effect on the ICER of vaccination and screening compared to screening only.

Finally, as shown, the vaccine remains cost effective (with QALYs as the outcome) if the age of screening can be delayed or a less frequent screening interval used.

## Discussion

These results suggest that adding vaccination to the current screening programme in the UK, to prevent infection with HPV types 6, 11, 16 and 18 is potentially cost-effective. The key parameters that affect this conclusion are the duration of vaccine efficacy and whether a booster is needed to achieve a duration that is sufficiently long to provide protection during the years of peak HPV incidence (modelled as a lifetime duration for this analysis). Our findings are consistent with previous analyses performed in the US that show that duration affects the cost-effectiveness of screening and vaccination compared to screening only [24,30]. However, in contrast to these analyses, our results suggest that a vaccination programme added to screening in the UK would be cost-effective without the need to change screening interval and/or the age of first screen. In the UK, screening is started at a later age, and a less frequent screening interval is used, compared to the US. The current UK strategy thus avoids the increased costs associated with detecting HPV-related changes, especially in younger women, that are more likely to regress.

To date, all cost-effectiveness analyses of HPV vaccination show that duration of efficacy will be a key to determining how cost-effective the vaccine will be. The need for a long duration of vaccine efficacy is consistent with our understanding of the natural history of HPV infection: progression to cervical cancer can take more than 10 years [41]. Currently, there is approximately 5 years of data of vaccine duration [42]. Long term monitoring of women currently participating in the vaccine trial will be needed to determine if and when a booster should be given. If a booster is needed our analyses show that the coverage achieved with a booster will affect the overall cost-effectiveness of vaccination and screening compared to screening only. One possible solution for increasing booster coverage beyond the 50% we modelled is if vaccination could be administered during the cervical cancer screening visit.

We did not use a quadrivalent type-specific model for this analysis. There is a need for population-based data that accounts for the distribution of these specific types within CIN from the UK. In addition, data on the impact of the vaccine on the overall reduction in CIN (as opposed to the type-specific reduction in CIN reported to date) due to these specific types, in previously unexposed girls, is also needed, to confirm the pooled estimates reported in the literature. Work is currently underway to refine existing models, including the one used here, to more accurately reflect the expected type-specific reduction in CIN and cancer when girls are vaccinated using data from the UK (Dr R. Barnabas, personal communication, 2007).

The use of QALYs is important since it allows us to incorporate, among other things, feelings of anxiety and embarrassment due to abnormal Pap test results as well as genital warts. However, the utilities used were derived from a study conducted among college-aged students in the US [39]. Although utilities derived from a UK population as well as a study of time spent in a given health state would more accurately reflect the morbidity associated with cancer, CIN and warts, this information has yet to be published. Results from the sensitivity analysis show that duration of symptoms has only a modest influence on the results.

Our model is conservative in that it does not take into account the impact of the vaccine on herd immunity. Prior analyses in the US with transmission models that accounted for herd immunity effects [43,44] suggest that the ICER for vaccination and screening compared to screening only would be much more attractive, even if the vaccine was only given to girls. In future analyses, we will also need to determine whether vaccinating boys in addition to girls will be cost-effective, taking into account the potential benefit of the HPV 6 and 11 component of the vaccine in preventing genital warts in men.

Although the vaccine has recently been approved for use in the UK, its use is not mandatory [4]. In addition, there has been no decision made on the choice of vaccine (Cervarix or Gardasil). As such, patients and payers will have to decide whether the cost of the vaccine represents value for money. To the extent that one vaccine has a higher cost than the other, and is not covered by a national program, vaccine coverage will differ from what we have modeled. Our analyses suggest that although there has not been a move to change screening to offset the costs of adding a vaccination program, one potential benefit of the vaccine that may make it more attractive for both patients and payers, is if eventually, a successful vaccination program allows women to be screened less frequently. As shown, depending on the characteristics of the vaccine, the age and/or frequency of screening may be delayed and still be cost-effective.

Other limitations include lack of a probabilistic sensitivity analysis and the fact that the model provides a conservative estimate of the true value of a quadrivalent HPV vaccine targeted at HPV types 6, 11, 16 and 18, in terms of health benefits as it does not take into consideration the potential reduction of adenocarcinoma, vulvar and vaginal intraepithelial neoplasia, vulval and vaginal cancers, as well as laryngeal papillomatosis associated with the vaccine HPV types [45]. In terms of the latter, the benefits of a quadrivalent vaccine are to some extent underestimated in this analysis. In terms of the former, although one study to date has conducted a probabilistic sensitivity analysis to determine credible intervals for the natural history component of the model [46], there is a lack of information to determine the appropriate distributions for use in models this complex. As such, this analysis used triangular distributions, although these have well known limitations. This highlights the need for epidemiologic studies to include information on the distributions as well as point estimates and confidence intervals.

## Conclusion

In conclusion, our results suggest that adding a quadrivalent vaccine to the current screening programme in the UK is potentially cost-effective. In order to more accurately quantify the effect that the vaccine will have, future models will need to account for the actual reduction in CIN and cancer based on data from the vaccine trials conducted in the UK, as well as to incorporate herd immunity effects.

## Competing interests

For this project, Dr. Kulasingam and Dr Myers have been supported by a grant from SP-MSD. Dr. Kulasingam and Dr Myers have been supported by grants from Merck and CSL-Australia. Dr. Kulasingam has been a consultant for SP-MSD and CSL-New Zealand. Nathalie Largeron is an employee of SP-MSD. Steve Bernard is a former employee of SP-MSD. Dr. Ruanne Barnabas has been a consultant for SP-MSD.

## Authors' contributions

SLK was responsible for the adaptation of a previously developed model used for this analysis, the analysis of the data, interpretation of results and drafting of the manuscript. SB was responsible for providing data and reviewing the manuscript. RVB was responsible for providing data and reviewing the manuscript. NL was responsible for providing data, reviewing the manuscript and providing independent confirmation of the cost-effectiveness results. EM was responsible for developing the original model used for this analysis, providing supervision for the conduct of the study and reviewing the manuscript. All authors have read and approved the final manuscript.
